# Supplementary material for: The interactome of intact mitochondria by cross-linking mass spectrometry provides evidence for coexisting respiratory supercomplexes
Source: Mol Cell Proteomics. 2017 Dec 8;17(2):216–32. doi: 10.1074/mcp.RA117.000470 (PMC5795388; doi:10.1074/mcp.RA117.000470)
Supplement: Supplemental Data [file supp_RA117.000470_133922_0_supp_23978_fzffwf.zip › spectra_annotation/mito_DR_spectra_annotation/436-1-49-1-67-1.pdf]

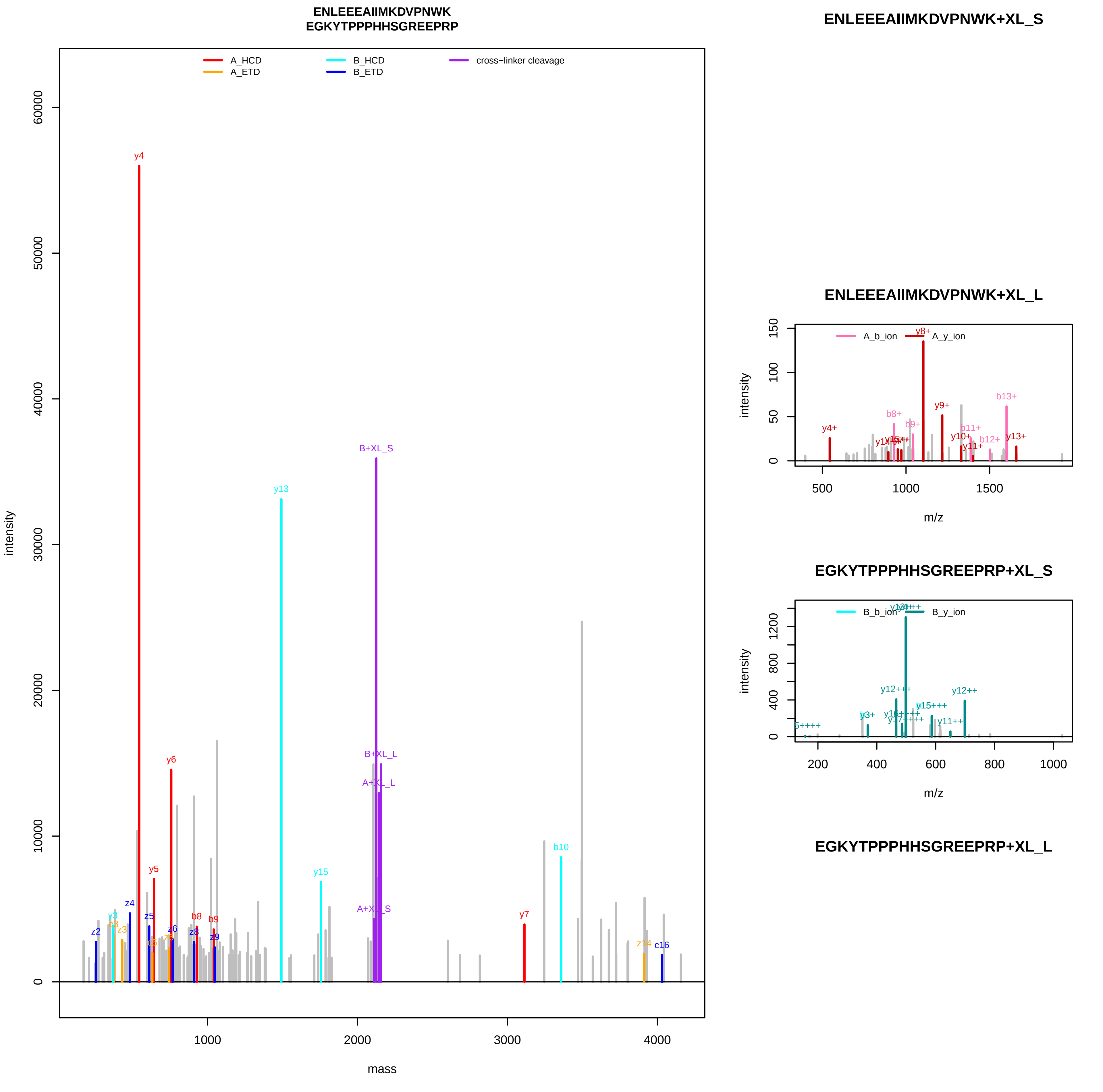

ENLEEEAIIMKDVPNWK
EGKYTPPPHHSGREEPRP
A_HCD
A_ETD
B_HCD
B_ETD
cross−linker cleavage
intensity
mass
0
10000
20000
30000
40000
50000
60000
1000
2000
3000
4000
y4
y6
y5
y13
y15
B+XL_S
B+XL_L
A+XL_L
A+XL_S
y7
b10
z14
c16
b8
b9
z2
z4
z5
z6
z8
z9
z3
ENLEEEAIIMKDVPNWK+XL_S
ENLEEEAIIMKDVPNWK+XL_L
A_b_ion
A_y_ion
m/z
y4+
y8+
y9+
b8+
b9+
b11+
b12+
b13+
y10+
y11+
y13+
EGKYTPPPHHSGREEPRP+XL_S
B_b_ion
B_y_ion
y12+++
y12++
y15+++
y3+
y11++
EGKYTPPPHHSGREEPRP+XL_L
